# Chapter 9: Analyses Using Disease Ontologies

**DOI:** 10.1371/journal.pcbi.1002827

**Published:** 2012-12-27

**Authors:** Nigam H. Shah, Tyler Cole, Mark A. Musen

**Affiliations:** Center for Biomedical Informatics Research, Stanford University, Stanford, California, United States of America; Whitehead Institute, United States of America; University of Maryland, Baltimore County, United States of America

## Abstract

Advanced statistical methods used to analyze high-throughput data such as gene-expression assays result in long lists of “significant genes.” One way to gain insight into the significance of altered expression levels is to determine whether Gene Ontology (GO) terms associated with a particular biological process, molecular function, or cellular component are over- or under-represented in the set of genes deemed significant. This process, referred to as enrichment analysis, profiles a gene-set, and is widely used to makes sense of the results of high-throughput experiments. The canonical example of enrichment analysis is when the output dataset is a list of genes differentially expressed in some condition. To determine the biological relevance of a lengthy gene list, the usual solution is to perform enrichment analysis with the GO. We can aggregate the annotating GO concepts for each gene in this list, and arrive at a profile of the biological processes or mechanisms affected by the condition under study. While GO has been the principal target for enrichment analysis, the methods of enrichment analysis are generalizable. We can conduct the same sort of profiling along other ontologies of interest. Just as scientists can ask “Which biological process is over-represented in my set of interesting genes or proteins?” we can also ask “Which disease (or class of diseases) is over-represented in my set of interesting genes or proteins?“. For example, by annotating known protein mutations with disease terms from the ontologies in BioPortal, Mort et al. recently identified a class of diseases—blood coagulation disorders—that were associated with a 14-fold depletion in substitutions at O-linked glycosylation sites. With the availability of tools for automatic annotation of datasets with terms from disease ontologies, there is no reason to restrict enrichment analyses to the GO. In this chapter, we will discuss methods to perform enrichment analysis using *any* ontology available in the biomedical domain. We will review the general methodology of enrichment analysis, the associated challenges, and discuss the novel translational analyses enabled by the existence of public, national computational infrastructure and by the use of disease ontologies in such analyses.

**What to Learn in This Chapter**

- Review the commonly used approach of Gene Ontology based enrichment analysis
- Understand the pitfalls associated with current approaches
- Understand the national infrastructure available for using alternative ontologies for enrichment analysis
- Learn about a generalized enrichment analysis workflow and its application using disease ontologies

This article is part of the “Translational Bioinformatics” collection for *PLOS Computational Biology*.

## 1. Introduction

Advanced statistical methods are most often used to perform the analysis of high-throughput data such as gene-expression assays [1]–[5], the result of which is a long list of “significant genes.” Extracting biological meaning from such lists is a nontrivial and time-consuming task, which is exacerbated by the inconsistencies in free-text gene annotations. The Gene Ontology (GO) offers a taxonomy that provides a mechanism to determine statistically significant functional subgroups within gene groups. One way to gain insight into the biological significance of alterations in gene expression levels is to determine whether the GO terms associated with the particular biological process, molecular function, or cellular component are over- or under-represented in the set of genes deemed significant by the statistical analysis [6]. This process, often referred to as “enrichment analysis,” can be used to summarize a gene-set [7], although it can also be relevant for other high-throughput measurement modalities including proteomics, metabolomics, and studies using tissue-microarrays [8].

With the availability of tools for automatic ontology-based annotation of datasets with terms from biomedical ontologies besides the GO, we need not restrict enrichment analysis to the GO. In this chapter, we outline the methodology of enrichment analysis, the associated challenges, and discuss novel analyses enabled by performing enrichment analysis using disease ontologies. We first review the current methods of GO based enrichment analysis to provide a foundation for discussing analyses using Disease Ontologies. Note that there is also research underway on the use of “pathways” for enrichment analyses as well as comparing statistically significant, concordant differences between two biological states as in Gene Set Enrichment Analysis [9], which are not discussed here.

### 1.1 Gene Ontology Enrichment Analysis

The goal of enrichment analysis is to determine which biological processes (or molecular function) might be predominantly affected in the set of genes that were deemed interesting or significantly changed. The simplest approach is to calculate functional ‘enrichment/depletion’ for each GO term—a higher (or lower) proportion of genes with certain annotations among the significantly changed genes than among all of the genes measured in the experiment. The finding of enrichment should not be interpreted as evidence implicating the GO term in the process studied without an appropriate statistical test.

The calculation of GO based functional enrichment involves two sets of items (usually genes or proteins): 1) The reference set, which is the set of items with which the “significant-set” is to be compared; the reference set may comprise all of the genes in the genome or all of the genes for which there were probes in the high throughput experiment; 2) The set of interest, which is the subset or list of significant genes that is to be analyzed for enrichment (or depletion) of GO terms in their annotations.

**Figure 1:**
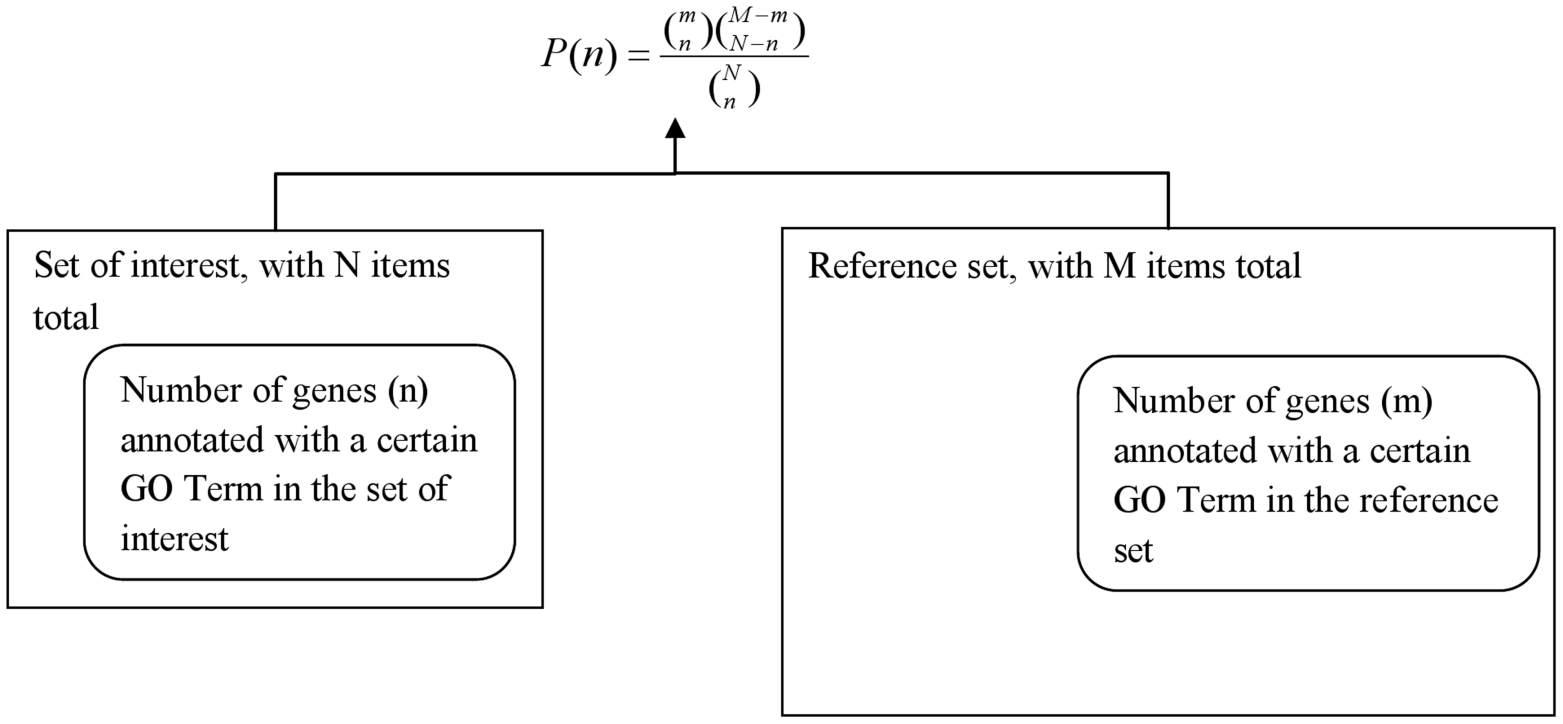
An overview of the process to calculate enrichment of GO categories. The steps usually followed are: (1) Get annotations for each gene in reference set and the set of interest. (2) Count the occurrence (n) of each GO term in the annotations of the genes comprising the set of interest. (3) Count the occurrence (m) of that same GO term in the annotations of the reference set. (4) Assess how “surprising” is it to find n, given m, M and N.

The analysis process (Figure 1) counts the GO annotations for both gene lists to calculate the number of genes (*n* and *m*) annotated with a particular GO term in each list and then calculates the probability (p-value) of the occurrence of at least *n* genes belonging to that category among the N genes in the set of interest, given that *m* genes are annotated with that term among the M genes in the reference set.

There are multiple ways to calculate the probability of observing a specific enrichment value. The simplest approach is to use a binomial model. For example, if one assumes that the probability of picking a gene annotated with the GO term ‘apoptosis’ is fixed and is equal to the proportion of genes annotated with ‘apoptosis’ in the reference set, then the binomial distribution provides the probability of obtaining a particular proportion of apoptosis genes among the genes in the set of interest by chance [10]. Such an approximation is quite reasonable for large reference sets (e.g. the whole genome) because the probability of selecting a gene annotated with the term ‘apoptosis’ into the set of interest does not change significantly after each selection.

However, when a gene or protein is picked from a smaller reference set, then the probability that the next picked gene is annotated to apoptosis is affected by whether the previously picked genes were annotated to apoptosis. Under these circumstances, the hypergeometric distribution—a discrete probability distribution that describes the number of successes in a sequence of n draws from a finite population without replacement—is a better statistical model. Another option is the Fisher's exact test or the chi-squared distribution, both of which take into consideration how the probabilities change when a gene is picked. The hypergeometric p-value is calculated using the following formula:
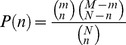
The p-value reports the likelihood of finding *n* genes annotated with a particular GO term in the set of interest by chance alone, given the number of genes annotated with that GO terms in the reference set. A biological process, molecular function or cellular location (represented by a GO term) is called enriched if the p-value is less than 0.05. GO annotations form the corner-stone of enrichment analysis in sets of differentially expressed genes. The GO project's Web site lists over 50 tools that can be used in this process [11].

Enrichment analysis can be done as a hypothesis-generating task, such as asking which GO terms are significant in a particular set of genes or a hypothesis-driven task such as asking whether apoptosis is significantly enriched or depleted in a particular set of genes.

In the hypothesis-driven setting, the analysis can include all of the genes that are annotated both directly to apoptosis and to its child nodes to maximize the statistical power because no correction for multiple comparisons is required. The hypothesis-generating approach allows an unbiased search for significant GO annotations. The analysis can be done with a bottom-up approach where for every leaf term the genes annotated with that GO term are also assigned to its immediate parent term. One can propagate the annotations recursively up along parent nodes until a significant node is found or until the root is reached. (Note: this upward propagation of annotations is referred to as computing the transitive closure of the annotation set over the graph of the Gene Ontology). Newer approaches can also perform the enrichment analysis accounting for the position of the term in the GO hierarchy [12]–[14].

#### 1.1.1 Interpretation of p-values

The p-values should be interpreted with caution because the choice of the reference set to which the set of interest is compared affects the p-value. For whole genome arrays, using the list of all genes on the array as the reference set is equivalent to using the complete list of genes in the genome. However, for arrays containing a selected subset of genes associated with a biological process, the choice of the gene set to use as the reference set is not obvious. Moreover, the p-value calculation using the hypergeometric distribution assumes the independence of the GO annotation categories, an assumption that may not be justified.

Another difficulty in determining significance using the calculated p-value and a cutoff of 0.05, especially in the hypothesis-generating approach mentioned above, is that multiple testing increases the likelihood of obtaining what appears to be a statistically significant value by chance. Multiple testing occurs because the GO term to be tested for enrichment is not pre-selected, but each term is tested. This allows multiple opportunities (equal to the number of terms tested) to obtain a statistically significant p-value by chance alone in a given gene list. However, correcting for multiple testing by using a Bonferroni correction in which the critical p-value cut-off is divided by the number of tests performed is too restrictive—especially when annotations are propagated up to the root node via a transitive closure; then the number of tests is equal to the number of terms in the GO hierarchy.

In this situation, calculation of the false discovery rate (FDR), which provides an estimate of the percentage of false positives among the categories considered enriched at a certain p-value cutoff, allows for a more informed choice of the p-value cutoff. One can estimate the false discovery rate (FDR) for the enriched categories by performing simulations which generate a user-specified number of random gene sets of the same size as the set of interest and calculate the average number of categories that are considered enriched in the random gene sets, at a p-value cutoff of 0.05. If the FDR is above the desired threshold, we can lower the p-value cutoff in order to re-duce the FDR to acceptable levels. Multiple hypothesis testing is a general problem that is not specific to GO (see [15] for a general review).

A related issue arising from performing the transitive closure—the propagation of annotations along the parent-child paths—is that the parallel tests performed for nodes in a given path will be correlated because the same genes can appear several times on each path. Correction methods that assumes independence of categories might not function well in this situation and might preclude identification of some categories that are indeed enriched [6]. It is possible to use the structure of the GO to decorrelate the analysis of various terms [12]–[14] or to use corrections methods such as a Benjamini–Yekutieli correction, which accounts for the dependency between the multiple tests [16].

### 1.2 Summary of Existing Limitations

In 2005, Khatri and Draghici noted that, despite widespread adoption, GO-based enrichment analysis has intrinsic drawbacks [17] and scientists must still rely on literature searches to understand a set of genes fully. These drawbacks represent conceptual limitations of the current state of the art and include:

- Incomplete annotations—even today, roughly 20% of genes lack any GO annotation
- Annotation bias because of inter-relationships between annotations (e.g. annotation with certain GO terms is not conditionally independent).
- Lack of a systematic mechanism to define a level of abstraction, to compensate for differing levels of granularity.

The remainder of the chapter discusses approaches to using existing, public bioinformatics tools to address these limitations and use disease ontologies in such analyses.

## 2. Using Disease Ontologies—Going beyond GO Annotations

As we have discussed, enrichment analysis provides a means of understanding the results of high-throughput datasets [17], [18]. Conceptually, enrichment analysis involves associating elements in the results of high-throughput data analysis to concepts in an ontology of interest, using the ontology hierarchy to create a summarization of the result, and computing statistical significance for any observed trend. The canonical example of enrichment analysis is in the interpretation of a list of differentially expressed genes in some condition. The usual approach is to perform enrichment analysis with the GO [17]. There are currently over 400 publications on methods and tools for GO-based enrichment, but (to the best of our knowledge) only a single tool, *Genes2Mesh*, uses something other than the GO (i.e. the Medical Subject Headings or MeSH), to calculate enrichment [19].

While GO has been the principal target for enrichment analysis, we can carry out the same sort of profiling using Disease Ontologies. Just as scientists can ask “*Which biological process is over-represented in my set of interesting genes or proteins?*”, they also should be able to ask “*Which disease (or class of diseases) is over-represented in my set of interesting genes or proteins?”* For example, by annotating known protein mutations with disease terms from the ontologies in BioPortal, Mort et al. recently identified a class of diseases—blood coagulation disorders—that were associated with a 14-fold depletion in substitutions at O-linked glycosylation sites [20].

There are several resources that can be used as disease ontologies for enrichment analysis. We use the term “disease ontology” to refer to artifacts—terminologies, vocabularies as well as ontologies—that can provide a hierarchy of parent-child terms for disease conditions. One of the most elaborate ontology for diseases is the Systematized Nomenclature for Medicine-Clinical Terms (SNOMED CT) is considered to be the most comprehensive, multilingual clinical healthcare terminology in the world [21]. SNOMED CT was a joint development between the NHS in England and the College of American Pathologists (CAP). It was formed in 1999 by the convergence of SNOMED RT and the United Kingdom's Clinical Terms Version 3 (formerly known as the Read Codes). As of 2007, SNOMED CT is maintained and distributed by the International Health Terminology Standards Development Organization (IHTSDO). Currently, SNOMED CT contains more than 311,000 active concepts with unique meanings and formal logic-based definitions organized into multiple hierarchies. The disease hierarchy is available under the clinical finding root node (analogous to the “biological process” root node in the Gene Ontology). Another widely used disease ontology is the National Cancer Institute thesaurus (NCIt), which is an ontology that provides terms for clinical care, translational and basic research, and public information and administrative activities. NCIt is a widely recognized standard for biomedical coding and reference, used by a variety of public and private institutions including the Clinical Data Interchange Standards Consortium Terminology (CDISC), the U.S. Food and Drug Administration (FDA), the Federal Medication Terminologies (FMT), and the National Council for Prescription Drug Programs (NCPDP). The disease hierarchy is available under the root node of “Diseases, Disorders and Findings”. The most widely *used* disease ontology is the International Classification of Diseases (ICD), which is part of the WHO Family of International Classifications. Version 9 of ICD is widely used in the United States for billing purposes in the health care system. Finally, there is effort to create an ontology of Human Diseases (available at http://diseaseontology.sourceforge.net) that conforms to the principles of the Open Biomedical Ontologies Foundry [22]. The Human Disease ontology is under review by the OBO Foundry since 2006. For the purpose of the current discussion, and enrichment analysis in general, pretty much disease ontology that provides a clear hierarchy of parent-child for diseases would be suitable for use.

Enrichment analysis owes its popularity to the fact that the process is methodologically straightforward and yields these easily interpretable results. Apart from analyzing results of high throughput experiments, enrichment analysis can also be used as an exploratory tool to generate hypotheses for clinical research. Computationally generated annotations (from multiple ontologies) on patient cohorts can provide a foundation for enrichment analysis for risk-factor determination. For example, enrichment analysis can identify general classes of drugs, diseases, and test results that are commonly found in readmitted transplant patients but not in healthy recipients. As noted, the GO has been the principal target for such analysis and despite widespread adoption, GO-based enrichment analysis has intrinsic drawbacks—the primary ones being incompleteness of and bias among available manually created annotations. Below, we discuss recent advances in the use of ontologies for automated creation of annotations that allow us to address these drawbacks and apply enrichment analysis using disease ontologies.

### 2.1 Advances in Ontology Access and Automated Annotation

There are several recent advances that enable us to use disease ontologies in enrichment analysis. The most obvious advancement is that almost all biomedical ontologies are now available in public repositories such as BioPortal [23]—built as a part of the NIH's Biomedical Information Science and Technology Initiative—which enables the use of terms from multiple ontologies in data analysis workflows. As of this writing, the BioPortal library contains more than 204 publicly accessible biomedical ontologies and their metadata, ranging in domains from genomics to clinical medicine to biomedical software resources, and comprising nearly 1.5 million terms. BioPortal's ontology library includes ontologies that individual investigators submit directly to BioPortal, terminologies drawn from both the Unified Medical Language System (UMLS) and the WHO Family of International Classifications (WHO-FIC). The BioPortal library also includes the ontologies that are candidates to the OBO Foundry, which is an initiative to create a set of well-documented and well-defined reference ontologies that are designed to work with one another to form a single, non-redundant system [22]. In addition to ontologies, BioPortal contains more than 1 million mappings between similar terms in different ontologies and 16.4 billion automatically created annotations on records from 22 public databases of biomedical data. Resources such as BioPortal provides a unified view of all its ontologies, which may be encoded in different formats, each of which has its own purpose, scope, and use. The unified view of the content enables uniform programmatic access to all ontologies and terminologies in the library for use in data analysis workflows.

The availability of automated annotation tools, such as the Annotator Web service from the NCBO and MetaMap from the National Library of Medicine allows the creation ontology-based annotations from free-text descriptions of gene and protein functions (such as GeneRIFs); as a result the lack of preexisting, manually assigned annotations is no longer a bottleneck. For example, the Annotator Web service enables users to provide a textual metadata of an item of interest—such as a GeneRIF describing a gene's function or an abstract corresponding to a PubMed record—to computationally generate ontology-based annotations for the item of interest. The user specifies which ontologies to use, and whether also to use mappings to other ontologies or transitive closure of hierarchy relations to extend the annotations. The service returns the ontology terms that it recognizes from the text—the annotations—and their position in the submitted record.

Finally the availability of large annotation repositories such as the Resource Index, which is a large repository of automatically created annotations by the NCBO, and the NIF database index, which is another large repository of computationally generated annotations on public data sources relevant to neuroscience, provide a source of co-occurrence statistics among ontology-terms in annotations. The availability of such annotation corpora makes the dependence between annotations with different ontology terms explicit.

Given these publicly available sources for ontologies, tools for creating ontology-based annotations and large repositories (or corpora) of annotations, it is now feasible to use disease ontologies in enrichment analysis in a manner similar to the Gene Ontology.

As we have discussed, one key aspect of calculating statistical enrichment is the choice of a reference-term frequency. It is not clear what the appropriate reference-term frequency should be when calculating enrichment of ontology-terms for which a “background set” is not defined. For example, in the case of Gene Ontology annotations, the background set is usually the GO annotations of the set of genes on which the data were collected or the GO annotations of a set of genes known in the genome for the species on which the data were collected. A background set is not available, however, when calculating enrichment using disease ontologies that have not been used for manual annotation in a way the Gene Ontology has. For this situation, there are two main options: 1) to construct a reference set programmatically (discussed in Section 2.3); or 2) use the frequency of particular terms in a large corpus, such as the Resource Index, Medline abstracts or on Web pages indexed by Internet search engines such as Google.

Multiple hypothesis testing—because each term is tested for enrichment individually—is also unavoidable when performing enrichment analysis with disease ontologies. However, methods of correcting the resultant increase in false discovery rates that work in the case of GO based enrichment analyses are directly applicable when using disease ontologies for such analyses.

Several researchers have noted that enrichment analysis is more meaningful when performed for combinations for terms [24]. For example, it is biologically more meaningful to know that a certain *molecular function* in a certain *biological process* at a certain *cellular location* is enriched than it is to know about each of the terms separately. Similarly, when using ontologies other than GO, it is more meaningful to look for enrichment of combinations such as certain adverse reactions in a given disease when treated by a particular drug. However, exhaustively examining all possible 3-term combinations of ontology terms is computationally expensive and most of the random term combinations make no biological sense. The identification of combinations that are meaningful and appear at a high enough frequency to justify their use in enrichment computations is an exciting and fruitful area of research.

### 2.2 DIY Disease Ontology-based Enrichment Analysis Workflow

**Figure 2:**
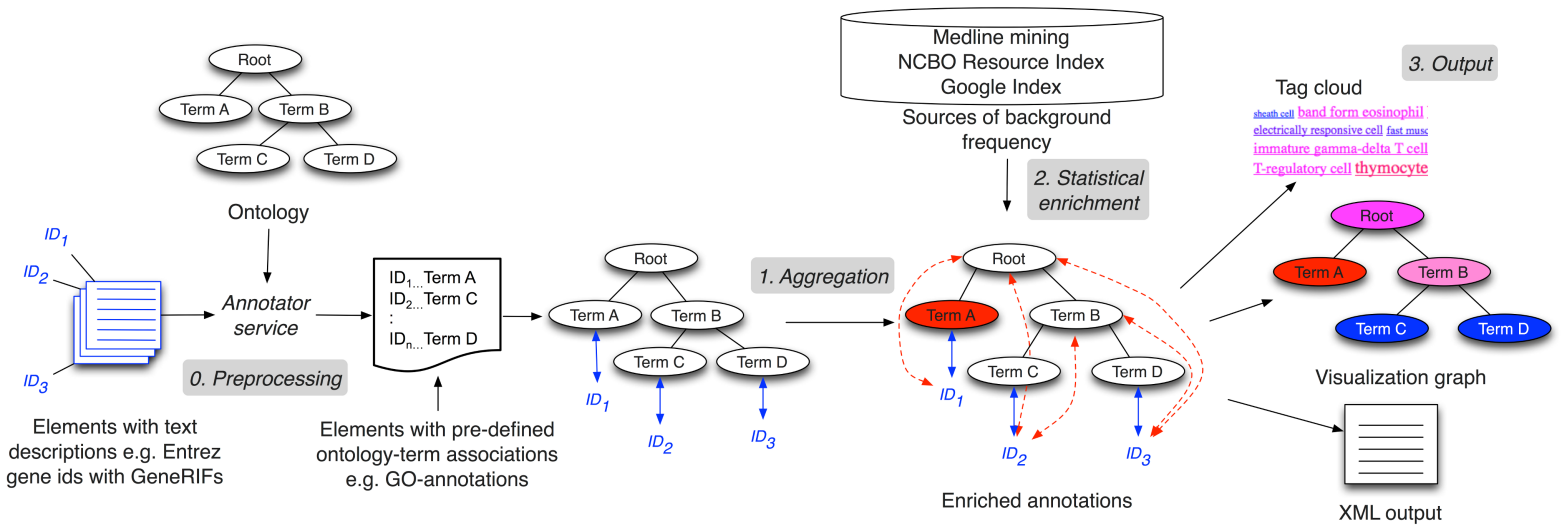
Workflow schematic of enrichment analysis. If the input set has only textual annotations, we first run the Annotator service to create ontology-term annotations. The annotation counts in the input set are first aggregated along the ontology hierarchy and then compared with a background set for a statistically significant difference in the frequency of each ontology term. If a significant difference in the term frequency is found, that term is called “enriched” in the input set of entities. The results of the analysis are returned either as a tag-cloud, a graph, or as an XML output that users can process as required.

We have seen that the progress in the current state of the art in storing, accessing and using ontologies for annotation provides components that allow enrichment analysis when preexisting annotations do not exist; as in the case of disease ontologies. We now discuss a workflow to conduct enrichment analysis in domains beyond just expression analysis. A schematic of the workflow is shown in Figure 2.

A user can start with two principal types of inputs. In the first case, the user already has the elements of the dataset of interest annotated with specific ontology terms—i.e. the user already has a file associating element identifiers (gene names, patient ID numbers, etc.) with ontology term identifiers. In the second case, the user has associations of identifiers to textual descriptions instead of ontology terms. For example, a user might have a file associating gene IDs with their GeneRIF descriptions from NCBI. In this situation a user can invoke the NCBO Annotator service [25], [26] to process these textual descriptions and assign ontology terms to the element identifiers (*Step 0*). Given the user's selection of an ontology, the annotator processes the input text (say GeneRIFs) to identify concepts that match ontology terms (based on preferred names or synonyms). The implementation details and accuracy of the Annotator service are described in [25]. The result is a list of computationally annotated element identifiers based on the input textual description, and this output is equivalent to the first input type. Using this step, we're able to create ontology-based annotations from free-text descriptions. Thus, we are no longer reliant on the availability of exhaustive manually-curated annotations, such as those required with GO-based analyses.

*Step 1* After this optional preprocessing step, for each ontology term in the input dataset one can programmatically traverse the ontology structure and retrieve the complete listing of paths from the concept to the root(s) of the ontology using Web services [27]. A traversal through each of these paths, essentially recapitulates the ontology hierarchy. Each term along the path is associated as an annotation to that element identifier in the input dataset to which the starting term was associated with. This procedure of tracing terms back to the graph's root performs the transitive closure of the annotations over the ontology hierarchy. In essence, for each child-parent (IS_A) relationship, we generate the complete set of implied (indirect) annotations based on child-parent relationships, by traversing and aggregating along the ontology hierarchy.

*Step 2* Once the ontology terms and their aggregate frequencies in the input dataset are calculated, we arrive at the step of determining the meaning or significance of the results. Enrichment analysis with GO has benefited from the existence of a natural and easily defensible choice for a background set—all of the given organism's genes, all genes measured on the platform, etc. For most of the disease ontologies we consider, no such comprehensive distribution exists [28]; and as discussed before, for calculating statistical enrichment, we need the background term frequency to determine if the aggregate annotation counts after step 1 are “surprising” given the background. By leveraging existing projects and resources, there are several methods by which a user can address this problem. We discuss a couple of heuristic approaches to address this problem, and in Section 2.3 discuss a systematic process to create custom reference sets.

In the first approach, one can access a database of automatically created annotations over the entirety of MEDLINE abstracts and use these annotations source as an approximate proxy for the true “background distribution” frequency of a specific term. To generate the background frequency, for a given term *X*, we retrieve the text strings corresponding to its preferred name and all of its synonyms, and then add up the MEDLINE occurrence counts for each of these strings. We return this number (*m*) as well as the total number of entries in the MEDLINE annotation database (*M*). The fraction *m/M* then represents the background frequency of the term *X* in the annotated corpus. Using this frequency we can compute significant comparative over- or under-representation in the input dataset.

The second approach uses NCBO's Resource Index, which is a repository of automatically-created annotations. Access to the Resource Index allows a user to make the same sort of calculations as with the MEDLINE term frequencies, but also offers information on the co-occurrence of ontological terms in textual descriptions and annotations of datasets; enabling the user to quantify the degree to which terms are independent or correlated in the annotation space.

**Figure 3:**
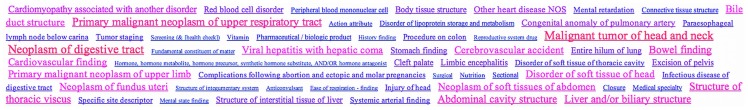
Tag cloud output: An example for the annotations of grants from FY1981 using SNOMEDCT. Blue denotes low-frequency terms and red denotes highly frequent terms. Many concepts, such as “neoplasm of digestive tract”, occur at high frequencies in most years, possibly denoting the constant focus on cancer research. An appropriate background term frequency distribution is necessary to determine significance of the high frequency.

*Step 3* There are several possible output mechanisms to such an analysis workflow. The simplest is a tag cloud, which intuitively summarizes the results of the analysis (Figure 3). The sizes and colors of terms in the cloud indicate the relative frequency of the terms offering a high-level overview. However, a tag cloud's representative ability is limited because there is no easy way to show significance relative to some expectation, or to show the elements in the input associated with some term.

**Figure 4:**
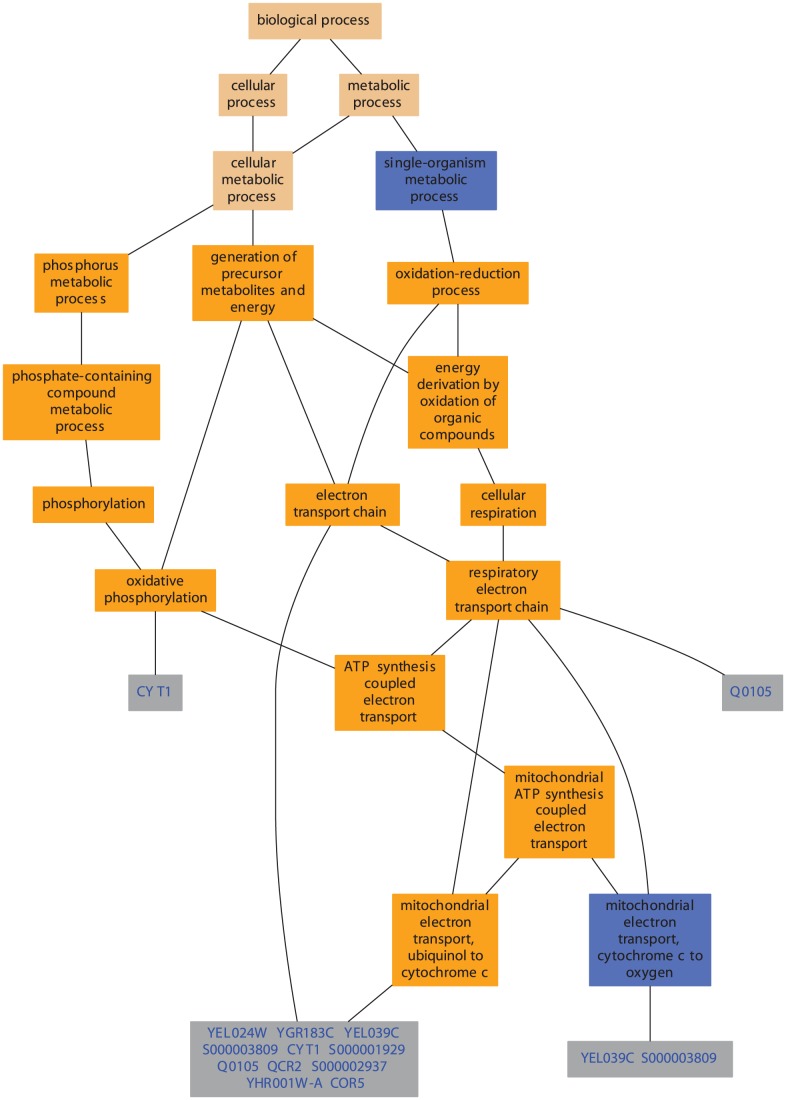
The figure shows a visualization generated using the GO TermFinder tool. The GO graph layout shows the significantly enriched GO terms in the annotations of the analyzed gene set. The color of the nodes is an indication of their Bonferroni corrected P-value (orange < = 1e-10; yellow 1e-10 to 1e-8; green 1e-8 to 1e-6; cyan 1e-6 to 1e-4; blue 1e-4 to 1e-2; tan >0.01).

The second output format is in XML, which is amenable to postprocessing by the user, as needed. The result for each term contains its respective frequency information in the input data along with the counts on which the frequency is based. The results on each term can also contain the list of identifiers that mapped to that term. Each node includes information on the level in the ontology at which the term is found. Using such an output, it is straight forward to create graphical visualizations similar to those that most GO based enrichment analysis tools provide [29]; see example in Figure 4.

#### 2.2.1. Ensuring quality

For any such custom analysis workflow it is essential to set up tests that ensure technical accuracy before interpreting the results for scientific significance. To evaluate technical accuracy, we suggest that users create benchmark data sets similar to those of Toronen and colleagues [30], who created gene lists with a selected enrichment level and a selected number of independent, over-represented classes to compare different GO-based enrichment methods. In the case of analyses using disease ontologies, the benchmark data sets would comprise gene lists enriched for specific disease terms, clinical-trial lists enriched for a specific drug being studied; lists of research publications that are enriched for known NCIt terms, and so on. A sample benchmark list of *aging* related genes and their annotations is provided in Section 5. Exercises. This dataset was compiled by computationally creating disease term annotations on 261 human genes designated to be related to aging according to the GeneAge database [31]. The annotations of this gene list are enriched for disorders, such as atherosclerosis, that are known to be associated with aging. Such benchmark data sets can be used to ensure accuracy of the enrichment statistics as well as to evaluate the appropriateness of different sources of reference-term frequencies for computing enrichment.

The inconsistency of abstraction levels in ontologies is an often discussed stumbling block for enrichment analysis [17]. Two terms at equal depths may not represent concepts of similar granularity, creating a bias in the reported term enrichment. By comprehensively analyzing the frequencies of terms in MEDLINE and the NCBO Resource Index, a user can perform a thorough analysis of dependencies among ontology-term annotations to make existing biases explicit as well as to define custom abstraction levels using methods developed by Alterovitz et al. [32]. The development of methods to reliably identify the appropriate level of abstraction at which to report the results of enrichment analysis is another exciting and fruitful area of research.

### 2.3 Creating Reference Sets for Custom Enrichment Analysis

As discussed before, a key pre-requisite for performing enrichment analysis is the availability of an appropriate reference dataset to compare against when looking for over- or under-represented terms. In this section, we describe: (i) a general method that uses hand-curated GO annotations as a starting point, for creating reference datasets for enrichment analysis using other ontologies; and (ii) a gene–disease reference annotation dataset for performing disease-based enrichment.

GO annotations are unique because highly trained curators associate GO terms to gene products manually, based on literature review. We describe how, with the availability of tools for automatic ontology-based annotation with terms from disease ontologies, it is possible to create reference annotation datasets for enrichment analysis using ontologies other than the GO—for example, the Human Disease Ontology.

Unlike GO terms, which actually appear in the text with low frequency, or gene identifiers, which are ambiguous, disease terms are amenable to automated, term extraction techniques. Therefore, using tools which recognize mentions of ontology terms in user submitted text, we can automatically recognize occurrences of terms from the Human Disease Ontology (DO) from a given corpus of text [28]; the key is to identify the text source that can be relied upon to recognize disease terms to associate with genes.

Unlike other natural-language techniques for finding gene–disease associations, our proposed method uses manually curated GO annotations as the starting basis to identify the text source from which to recognize disease terms. Basically, we use manually curated GO annotations to identify those publications that were the basis for associating a GO term with a particular gene.

**Figure 5:**
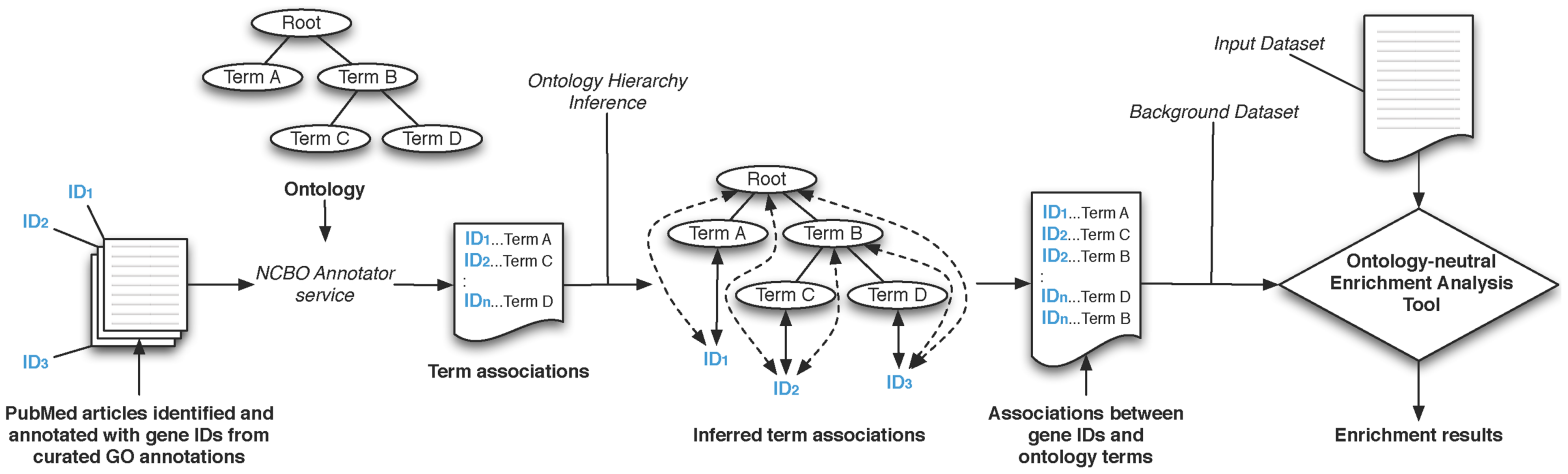
Workflow for generating background annotation sets for enrichment analysis: We obtain a set of PubMed articles from manually curated GO annotations, which we process using the NCBO Annotator service.

Figure 5 summarizes our method. First, we start with GO annotations, which provide the PubMed identifiers of papers based on which gene products are associated with specific GO terms by a curator. The annotations essentially give us a link between gene identifiers and PubMed articles and only those PubMed articles that were deemed to be relevant for GO annotation curation. Next, we recognize terms from an ontology of interest (e.g. Human Disease) in the title and abstracts of those articles. Finally, we associate the recognized ontology terms with the gene identifiers to which the article analyzed was associated.

In order to demonstrate feasibility of the proposed workflow and to provide a sample reference annotation set for performing disease ontology based analyses in the exercises of this chapter, we download GO annotation files for human gene products from geneontology.org. These files are tab-delimited text files that contain, among other things, a list of gene identifiers, associated GO terms, and the publication source (a PubMed identifier) on the basis of which that GO annotation was created. We removed all electronically inferred annotations (IEA) from the annotation file. We also removed all qualified annotations, such as negated (NOT) ones. As a result, we obtain a list of publications and the genes they describe, gene–publication tuples. In the next step, using the PubMed identifiers obtained from the GO annotation files, we fetch each article's title and abstract using the National Library of Medicine eUtils. We save each article's title and abstract as a file and annotate it via the Annotator service using the disease ontology as the target. Once we have the publication–disease tuples, we cross-reference them with the gene–publication tuples resulting in gene–disease associations for 7316 human genes.

Out of 25,000 currently estimated human genes, we are able to annotate 7316 genes (29.2%) with at least one disease term from the Human Disease Ontology. Previous methods that use advanced text mining have been able to annotate 4408 genes (17.7%) [33]. A study based on OMIM associated 1777 genes (7.1%) with disease terms to create a human “diseasome” [34] and an automated approach using MetaMap as the concept recognizer and GeneRIFs as well as descriptions from OMIM as the input textual descriptions annotated roughly 14.9% of the human genome with disease terms [28]. Because the number of human genes known at the time of each study varies, we make the comparisons loosely.

**Figure 6:**
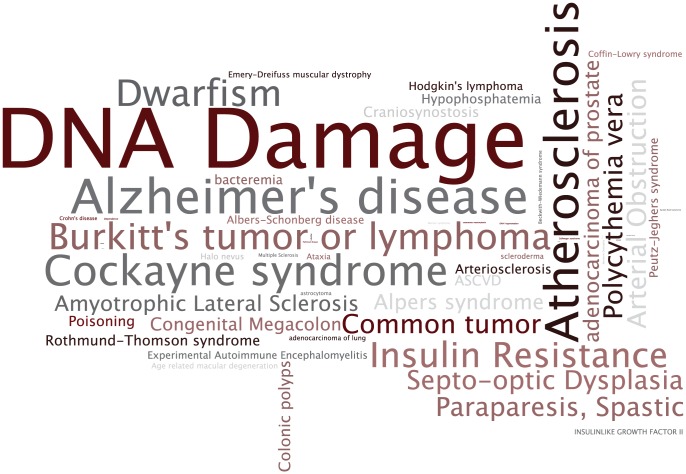
Disease terms significantly enriched in annotations of aging-related genes: The tag cloud shows those disease terms in the annotations of the 261 aging related genes that are statistically enriched given our gene–disease background annotation dataset. Terms that are significantly enriched appear larger. We used a binomial test to detect enriched disease terms in the aging related gene set. Note that mis-annotated terms (such as Recruitment) and non-informative terms (such as Disease) are not deemed enriched by the statistical analysis.

In order to validate our background annotation set, we evaluated our gene–disease association dataset in several ways described in [35]. First, we examined a set of genes related specifically to aging from the GenAge database [31] for their coherence in terms of the assigned disease annotations. Next, we performed disease-based enrichment analysis on the same aging related gene set using our newly created reference annotation set. The results of the enrichment analysis are shown in Figure 6 and the analysis itself is offered as an exercise for the reader in Section 6. Exercises. What differentiates our suggested method from other approaches [28], [36] for finding gene–disease associations is the use of GO annotations as a basis for identifying reliable gene–publication records that serve as the foundation for generating automated annotations. Furthermore, researchers can reuse our method to examine function along other dimensions. For example, researchers can use the Pathway ontology to generate gene–pathway associations.

#### 2.3.1 Ensuring quality

When using an automated annotation process to create a reference annotation set, there are some caveats to consider. First, not all ontologies are equally suited for creating automated annotations. Second, automated annotation depends highly on the quality of the input text corpus. Third, some errors in annotation are inevitable in an automated process. We discuss these issues below.

*Using other ontologies*. Although we specifically focus on creating annotations with terms from the human disease ontology, the method we have devised (Figure 6) can create annotations with terms from other ontologies. In the presented workflow, to obtain a background dataset for enrichment for some ontology other than DO, researchers would simply configure a parameter for the Annotator Web service to use their ontology of choice from BioPortal. In fact, other researchers have used a similar annotation workflow to recognize morphological features in textual descriptions of fish species [37].

Not all ontologies are viable candidates for automatic annotation because not all ontology terms appear in the text of a MEDLINE abstract. For example, using term–frequency counts in MEDLINE abstracts [38], we calculated that disease terms are mentioned 46% more often than GO terms in MEDLINE abstracts. As another example, only 10% of the manually assigned GO terms can be detected directly in the paper abstract supporting that particular GO annotation. Because disease terms are mentioned significantly more often than GO terms, the automated annotation process works well for annotating genes with disease ontology terms.

*Missing annotations*. Out of the 261 aging-related genes in our evaluation subset, the Annotator left out 24 genes (9%), for which we have no disease terms associated with those genes in our gene–disease association dataset. These missed annotations provide an opportunity for refining the annotation workflows to use sources of text beyond just the papers referenced in GO annotations.

*Annotation errors*. Some errors in annotation are inevitable in an automated process. For example, in the reference annotation set we created, TP53 was also annotated, wrongly, to “Recruitment”. Papers that were the basis of creating GO annotations for TP53 certainly mention the term “Recruitment”; however that term is not a *disease*. The term “Recruitment” is in the Human Disease Ontology and is declared to be a synonym of “auditory recruitment”, which does not have an asserted superclass, or a place in the hierarchy indicating a possible error in the ontology. However, because such errors will affect annotation of both the set of interest and the reference set equally, the errors will most likely cancel each other out when computing statistical enrichment (Figure 6)—though that is not guaranteed. Advanced text mining can potentially provide checks against such kinds of errors by analyzing the context in which a potential disease term is mentioned.

## 3. Novel Use Cases Enabled

We believe that extending the current enrichment-analysis methods to ontologies beyond GO and to extending the method beyond analyzing gene and protein annotations to any set of entities for term enrichment will enable several novel use cases. For example, a user might analyze a set of papers published in the last three years in a particular domain (say, signal transduction) and identify which pathway was mentioned most frequently. Similarly, a user could analyze descriptions of genes controlled by a particular ultra-conserved region of DNA to generate hypotheses about the region's function in specific disease processes. We discuss the potential of some of the novel use cases enabled by disease ontology based enrichment analysis.

*Analysis of protein annotations* To demonstrate the feasibility of performing enrichment analysis and recovering known GO annotations as well as to demonstrate enrichment analysis with multiple ontologies, we analyzed a list of 261 known aging related genes from the GenAge database [31]. We started by collecting textual descriptions for UniProt protein entries corresponding to each human gene in the GenAge database. The textual descriptions included the protein name, gene name, general descriptions of the function and catalytic activity as well as keywords and GO terms. We processed this text as described in the workflow in Figure 6 and created annotations from Medical Subject Heading (MSH), Online Mendelian Inheritance in Man (OMIM), UMLS Metathesaurus (MTH) and Gene Ontology (GO).

We created a background set of annotations on 19671 proteins by applying the same protocol to manually annotated and reviewed proteins from SwissProt (Jan 2010 version). We calculated enrichment and depletion of specific terms, corrected for multiple hypotheses and obtained a list of significant terms for all four ontologies. Not surprisingly, ‘aging’ is an enriched term. There were several other terms enriched such as ‘electron transport’ (2.79e-10), ‘protein kinase activity’ (2.8e-10) and ‘nucleotide excision repair’ (8.78e-07) which appeared in MSH, MTH, and GO. The enriched terms also included aging associated diseases such as ‘Alzheimer's disease’ (0.01), ‘Werner syndrome’ (5.3e-05), ‘Diabetes Mellitus’ (1.5e-04) and ‘neurodegeneration’ (2.5e-03) from OMIM.

This case study demonstrate that enrichment analysis with multiple ontologies is feasible and it enables a comprehensive characterization of the biological “signal” present in gene/protein lists [39]. For example, by annotating known protein mutations with disease terms from the ontologies in BioPortal, Mort et al. recently identified a class of diseases—blood coagulation disorders—that were associated with a 14-fold depletion in substitutions at O-linked glycosylation sites [20].

*Analysis of funding trends* To demonstrate the feasiblity of such analyses in a novel domain, we processed the funding allocations of the NIH in fiscal years 1980–1989. We aimed to identify trends in institutional funding priorities over time, as represented by changes in the relative frequencies of ontology concepts from year-to-year. Using a database containing the complete set of grants in this interval—with their titles, amounts, recipient institutions, etc.—we selected grants worth over $1,250,000 (in constant 2008 dollars). We annotated the titles of these grants with SNOMEDCT terms and used the annotation sets to generate tag clouds for each year, such as the one shown in Figure 3 for year 1981, to create a visual summary of funding trends on a per year basis. Further analysis cross-linking annotation on grants with annotations on publications from specific institutions can enable comparative analysis of the research efficacy at different institutions.

*Hypothesis generation for Clinical Research* Finally, enrichment analysis can also be used as an exploratory tool to generate hypotheses for clinical research by analyzing annotations on medical records in conjunction with annotation of molecular datasets. For example, in the case of kidney transplants, extended-criteria donor (ECD) organs have a 40% rate of delayed graft function and a higher incidence of rejection compared to standard-criteria donor (SCD) kidneys. Identifying causes of this difference is crucial to identify patients in which an ECD transplant has a high chance of working.

At several research sites, the datasets collected to address this question comprise immunological metrics beyond the standard clinical risk factors, including multi-parameter flow-cytometric analysis of the peripheral immune-cell repertoire, genomic analysis, and donor-specific functional assessments. These patient data sets can be annotated using automated methods [8], [26] to enable enrichment analysis for risk-factor determination.

For example, simple enrichment analysis might allow identification of classes of drugs, diseases, and test results that are commonly found only in readmitted transplant patients. Enrichment analysis to identify common *pairs* of terms of different semantic types can identify combinations of drug classes and co-morbidities, or test risk-factors and co-morbidities that are common in this population.

## 4. Summary

Because enrichment analysis with GO is widely accepted and scientifically valuable, we argue that the logical next step is to extend this methodology to other ontologies—specifically disease ontologies.

Given the recent advances in ontology repositories and methods of automated annotation, we argue that enrichment analysis based on textual descriptions is possible.

We have systematically discussed how to accomplish enrichment analysis using ontologies other than the Gene Ontology as well as address some of the limitations of existing analysis methods. For example, the roughly 20% of genes that lack annotations can now be associated, via their GeneRIFs, with terms from disease ontologies. We have outlined possible directions of research for overcoming other limitations such as inconsistent abstraction levels in ontologies, performing the analysis using combinations of ontology terms, and accounting for annotation bias.

In order to perform enrichment analysis using ontologies other than the GO, a key pre-requisite is the availability of a background set of annotations to enable the enrichment calculation. We have described a general method, which uses hand-curated GO annotations as a starting point, for creating background datasets for enrichment analysis using other ontologies—such as the Human Disease Ontology, for which hand-curated annotations are not available.

To demonstrate the feasibility and utility of our proposals, we have created a background set of annotations to enable enrichment analysis with the Human Disease Ontology and validated that background set by using the created annotations to examine the coherence of known aging related genes and by performing enrichment analysis on an aging related gene set from the GeneAge database [31]. We make the set of aging related genes and the reference annotation set available for reader exercises in enrichment analysis.

We argue that enrichment analysis using computationally created ontology-based annotations from textual descriptions is possible, thus introducing enrichment analysis as a research methodology in new domains such as hypothesis generation for clinical research; without requiring manually created annotations.

## 5. Exercises

(1) For the 260 aging related genes in Dataset S1, perform enrichment analysis using the Human Disease ontology, using Dataset S2 as the reference annotation set. Some considerations while working through the problem:

- The genes are listed with their UniprotIDs.
- Using the notation in Section 1.1, the values of *N* and *M* are the total number of unique genes in the aging set and total set, respectively, and *not* the number of unique terms. The values of *n* and *m* are the unique genes that are annotated with a given term in the corresponding set.
- When performing the hypergeometric test, if the test calculates the *p* value based on finding a value of *n* greater than or less than what was observed (instead of equal to what was observed), remember to add or subtract 1 from the number of genes annotated with a given term when calculating. If you are using a function to calculate, refer to the documentation to understand the input required.
- Consider from which tail of the hypergeometric distribution you wish to calculate the *p* value.

(2) For the 260 aging related genes, perform enrichment analysis using SNOMED-CT (Systematized Nomenclature of Medicine-Clinical Terms). Use the GeneRIF (Gene Reference into Function) database as the source text to annotate with disease terms from SNOMED-CT. Choose an appropriate reference annotation set and justify the choice. Some considerations while working through the problem:

- An index of GeneRIFs, maintained by the National Center for Biotechnology Information (NCBI) and the National Institutes of Health (NIH), can be downloaded from here: ftp://ftp.ncbi.nih.gov/gene/GeneRIF/
- Mapping from UniprotIDs to GeneIDs, which are used in the GeneRIF database, can be done here: http://www.uniprot.org/help/mapping. Note that you will get 261 GeneIDs for the 260 UniprotIDs.
- Annotation using the National Center for Biomedical Ontology's BioPortal Annotator Service requires obtaining an API key. This can be done after registration and going to “Account” where your API key will be displayed: http://bioportal.bioontology.org/
- Information on the programmatic use of the BioPortal Annotator as a client can be found here: http://www.bioontology.org/wiki/index.php/Annotator_Web_service. Example code from numerous languages, including Java, R, Python, Ruby, Excel, HTML, and Perl, can be found here: http://www.bioontology.org/wiki/index.php/Annotator_Client_Examples. All NCBO REST Web services require the parameter “apikey = YourApiKey”. It is strongly encouraged that all users of the NCBO Annotator Web service use only the **virtual ontology identifier**. To do so, set the “isVirtualOntolgyId” parameter to “true”. This will ensure that you access the version of the ontology that is actually in the database. Failure to do this will result in your code breaking every time the database is updated.
- Output from the annotation service can be conveniently parsed in XML. To see an example of what this might look like, visit http://bioportal.bioontology.org/annotator. Insert the sample text or use text of your choice, makes selection(s) under ‘Select Ontologies’ and ‘Select UMLS Semantic Types’ and click ‘Get Annotations’. At the bottom by ‘Format Results As’ you can select XML to see the XML tree structure of the Annotator output.
- The suggested ontology for this exercise is SNOMED-CT (ontology ID: 1353) and semantic types Anatomical Structure (T017), Disease or Syndrome (T047), Neoplastic Process (T191), and NCBO BioPortal concept (T999).
- Some processing of the GeneRIF text may be necessary to prevent errors in annotation. It is suggested to remove GeneRIFs with new line characters (‘\n’) and replace single or double quotes with white space.
- Many GeneIDs have multiple GeneRIF entries. The user will find more efficient annotation if all of the GeneRIF entries for a given gene are concatenated and passed to the annotator instead of annotating individual GeneRIF entries for the same gene.
- Due to the large number of GeneRIFs, the BioPortal Annotator may timeout while the user is looping through genes to annotate. It is suggested that the annotation is done incrementally and joined or intermittent saves of the annotations is done to prevent timely re-annotation.
- The given set of aging genes will have considerably more annotations terms per gene than the set of all genes in the GeneRIF database. This bias should be a consideration when deciding on an appropriate *M*. There are numerous approaches to address this, and a simple method may be to limit the reference set of genes *M* to only those with at least a given number of annotated terms. You may also want to limit the results to only those terms that appear at least a given amount of times in the aging gene annotations.

Answers to the Exercises can be found in Text S1.

**Further Reading**

- Tirrell R, Evani U, Berman AE, Mooney SD, Musen MA, et al. (2010) An ontology-neutral framework for enrichment analysis. AMIA Annual Symposium proceedings/AMIA Symposium AMIA Symposium 2010: 797–801.
- Shah NH, Jonquet C, Chiang AP, Butte AJ, Chen R, et al. (2009) Ontology-driven indexing of public datasets for translational bioinformatics. BMC Bioinformatics 10 Suppl 2: S1.
- Alterovitz G, Xiang M, Mohan M, Ramoni MF (2007) GO PaD: the Gene Ontology Partition Database. Nucleic Acids Res 35(Database issue): D322–D327.
- Myhre S, Tveit H, Mollestad T, Laegreid A (2006) Additional gene ontology structure for improved biological reasoning. Bioinformatics 22: 2020–2027.
- Toronen P, Pehkonen P, Holm L (2009) Generation of Gene Ontology benchmark datasets with various types of positive signal. BMC Bioinformatics 10: 319.

## Supporting Information

Dataset S1 Data file for Exercise 1
(TXT)

Dataset S2 Data file for Exercise 1
(TXT)

Dataset S3 Data file for Exercise 1
(OBO)

Dataset S4 Additional info on genes mentioned in S1 and S2. Can be used in lieu of GeneRIFs in Exercise 2.
(TXT)

Text S1 Answers to Exercises
(DOCX)

Table S1 Exercise 1 analysis results
(CSV)

Table S2 Exercise 2 analysis results
(CSV)
