# Supplementary material for: Chapter 9: Analyses Using Disease Ontologies
Source: PLoS Comput Biol. 2012 Dec 27;8(12):e1002827. doi: 10.1371/journal.pcbi.1002827 (PMC3531278; doi:10.1371/journal.pcbi.1002827)
Supplement: Text S1 — Answers to Exercises (DOCX) [file pcbi.1002827.s005.docx]

# Answers to Exercises

**(1)** For the 260 aging related genes in Dataset S1, perform enrichment analysis using the Human Disease ontology, using Dataset S2 as the reference annotation set. Some considerations while working through the problem:

- The genes are listed with their UniprotIDs.
- Using the notation in section 1.1, the values of *N* and *M* are the total number of unique genes in the aging set and total set, respectively, and *not* the number of unique terms. The values of *n* and *m* are the unique genes that are annotated with a given term in the corresponding set.
- When performing the hypergeometric test, if the test calculates the p value based on finding a value of n greater than or less than what was observed (instead of equal to what was observed) remember to add or subtract 1 from the number of genes annotated with a given term when calculating. If you are using a function to calculate, refer to the documentation to understand the input required.
- Consider from which tail of the hypergeometric distribution you wish to calculate the *p* value.

**Answer:** *For list of disease categories deemed enriched as answers to question 1, see Table S1.*

**(2)** For the 260 aging related genes, perform enrichment analysis using SNOMED-CT (Systematized Nomenclature of Medicine-Clinical Terms). Use the GeneRIF (Gene Reference into Function) database as the source text to annotate with disease terms from SNOMED-CT. Choose an appropriate reference annotation set and justify the choice. Some considerations while working through the problem:

- An index of GeneRIFs, maintained by the National Center for Biotechnology Information (NCBI) and the National Institutes of Health (NIH), can be downloaded from here: <ftp://ftp.ncbi.nih.gov/gene/GeneRIF/>
- Mapping from UniprotIDs to GeneIDs, which are used in the GeneRIF database, can be done here: <http://www.uniprot.org/help/mapping>. Note that you will get 261 GeneIDs for the 260 UniprotIDs.
- Annotation using the National Center for Biomedical Ontology’s BioPortal Annotator Service requires obtaining an API key. This can be done after registration and going to"Account" where your API key will be displayed: <http://bioportal.bioontology.org/>
- Information on the programmatic use of the BioPortal Annotator as a client can be found here: <http://www.bioontology.org/wiki/index.php/Annotator_Web_service>. Example code from numerous languages, including Java, R, Python, Ruby, Excel, HTML, and Perl, can be found here: <http://www.bioontology.org/wiki/index.php/Annotator_Client_Examples>. All NCBO REST Web services require the parameter "apikey=YourApiKey". It is strongly encouraged that all users of the NCBO Annotator Web service use only the **virtual ontology identifier**. To do so, set the "isVirtualOntolgyId" parameter to "true". This will ensure that you access the version of the ontology that is actually in the database. Failure to do this will result in your code breaking every time the database is updated.
- Output from the annotation service can be conveniently parsed in XML. To see an example of what this might look like, visit <http://bioportal.bioontology.org/annotator>. Insert the sample text or use text of your choice, makes selection(s) under ‘Select Ontologies’ and ‘Select UMLS Semantic Types’ and click ‘Get Annotations’. At the bottom by ‘Format Results As’ you can select XML to see the XML tree structure of the Annotator output.
- The suggested ontology for this exercise is SNOMED-CT (ontology ID: 1353) and semantic types Anatomical Structure (T017), Disease or Syndrome (T047), Neoplastic Process (T191), and NCBO BioPortal concept (T999).
- Some processing of the GeneRIF text may be necessary to prevent errors in annotation. It is suggested to remove GeneRIFs with new line characters (‘\n’) and replace single or double quotes with white space.
- Many GeneIDs have multiple GeneRIF entries. The user will find more efficient annotation if all of the GeneRIF entries for a given gene are concatenated and passed to the annotator instead of annotating individual GeneRIF entries for the same gene.
- Due to the large number of GeneRIFs, the BioPortal Annotator may timeout while the user is looping through genes to annotate. It is suggested that the annotation is done incrementally and joined or intermittent saves of the annotations is done to prevent timely re-annotation.
- The given set of aging genes will have considerably more annotations terms per gene than the set of all genes in the GeneRIF database. This bias should be a consideration when deciding on an appropriate *M*. There are numerous approaches to address this, and a simple method may be to limit the reference set of genes *M* to only those with at least a given number of annotated terms. You may also want to limit the results to only those terms that appear at least a given amount of times in the aging gene annotations.

**Answer:** *This is an open ended question and does not have a fixed answer. Depending on the date on which GeneRIFs are annotated using a specific version of SNOMEDCT, the background set generated will differ. The answer set provided in Table S2 are from 10/20/2012 done using SNOMEDCT version 2011_07_31.*
